# Biomarkers of Neurodegeneration and Alzheimer’s Disease Neuropathology in Adolescents and Young Adults with Youth-Onset Type 1 or Type 2 Diabetes: A Proof-of-Concept Study

**DOI:** 10.3390/endocrines5020014

**Published:** 2024-05-06

**Authors:** Allison L. B. Shapiro, Christina Coughlan, Brianne M. Bettcher, Meghan E. Pauley, Jeongchul Kim, Petter Bjornstad, Benjamin Rajic, Jennifer Truong, Christopher Bell, Ye Ji Choi, Keenan A. Walker, Huntington Potter, Angela D. Liese, Dana Dabelea, Christopher T. Whitlow

**Affiliations:** 1 Lifecourse Epidemiology of Adiposity and Diabetes Center, University of Colorado at Anschutz (CU-Anschutz), Aurora, CO 80045, USA;; 2 Section of Endocrinology, Department of Pediatrics, School of Medicine (SOM), CU-Anschutz, Aurora, CO 80045, USA;; 3 University of Colorado Alzheimer’s and Cognition Center, CU-Anschutz, Aurora, CO 80045, USA;; 4 Department of Neurology, SOM, CU-Anschutz, Aurora, CO 80045, USA;; 5 Barbara Davis Center for Diabetes, CU-Anschutz, Aurora, CO 80045, USA;; 6 Radiology Informatics and Image Processing Laboratory, Department of Radiology, Wake Forest School of Medicine, Winston-Salem, NC 27101, USA;; 7 Division of Renal Diseases and Hypertension, Department of Medicine, SOM, CU-Anschutz, Aurora, CO 80045, USA; 8 Laboratory of Behavioral Neuroscience, National Institute on Aging, Intramural Research Program, Baltimore, MD 20814, USA;; 9 Department of Epidemiology and Biostatistics, Arnold School of Public Health, University of South Carolina, Columbia, SC 29208, USA;

**Keywords:** youth-onset diabetes, Alzheimer’s disease, neurodegeneration, plasma biomarkers, amyloid, tau

## Abstract

Adult-onset diabetes increases one’s risk of neurodegenerative disease including Alzheimer’s disease (AD); however, the risk associated with youth-onset diabetes (Y-DM) remains underexplored. We quantified plasma biomarkers of neurodegeneration and AD in participants with Y-DM from the SEARCH cohort at adolescence and young adulthood (Type 1, n = 25; Type 2, n = 25; 59% female; adolescence, age = 15 y/o [2.6]; adulthood, age = 27.4 y/o [2.2]), comparing them with controls (adolescence, n = 25, age = 14.8 y/o [2.7]; adulthood, n = 21, age = 24.9 y/o [2.8]). Plasma biomarkers, including glial fibrillary acidic protein (GFAP), neurofilament light chain protein (NfL), phosphorylated tau-181 (pTau181), and amyloid beta (Aβ40, Aβ42), were measured via Simoa. A subset of participants (n = 7; age = 27.5 y/o [5.7]) and six controls (age = 25.1 y/o [4.5]) underwent PET scans to quantify brain amyloid and tau densities in AD sensitive brain regions. Y-DM adolescents exhibited lower plasma levels of Aβ40, Aβ42, and GFAP, and higher pTau181 compared to controls (*p* < 0.05), a pattern persisting into adulthood (*p* < 0.001). All biomarkers showed significant increases from adolescence to adulthood in Y-DM (*p* < 0.01), though no significant differences in brain amyloid or tau were noted between Y-DM and controls in adulthood. Preliminary evidence suggests that preclinical AD neuropathology is present in young people with Y-DM, indicating a potential increased risk of neurodegenerative diseases.

## 1. Introduction

Adult-onset diabetes is a significant risk factor for cognitive impairment and dementia [1–5]. However, despite a vast body of literature on the relationship between adult-onset diabetes and risk of cognitive impairment and dementia, and our growing understanding of the mechanistic links between diabetes pathophysiology and neurodegeneration and Alzheimer’s Disease (AD) neuropathology, significant knowledge gaps remain.

Youth-onset diabetes has been overlooked, with potentially devastating consequences for accelerated pathological aging in young people with type 1 diabetes (T1D) or type 2 diabetes (T2D). By the average age of onset for adult diabetes (~46 years-old [6]), people with youth-onset diabetes have lived with their disease for well over 30 years. Importantly, a recent prospective population-based cohort in the United Kingdom found that a younger age at onset of diabetes corresponded to a younger age at onset of dementia [7]. While this study did not include people with youth-onset diabetes, its findings added to the growing body of evidence implicating the timing of diabetes onset and the duration of the disease as potent contributors to an earlier onset and higher incidence of dementia [8–11]. From these data, it may follow that people with youth-onset diabetes, whose diabetes onset occurs early in the life course and whose disease duration is at least twice that of people with adult-onset diabetes, are at a substantial risk of premature development of cognitive impairment and dementia with possible AD neuropathology.

Few studies have investigated the long-term impact of youth-onset diabetes on the risk for, and timing of, developing cognitive impairment and preclinical signs of AD neuropathology. Compared to people of a similar age who do not have diabetes, people with adult-onset diabetes have a 60–80% greater likelihood of incident AD [3,5,12–17]. The existing studies in youth-onset diabetes and AD are limited to middle-age and older adults with T1D, and most lack measures of neuropathology [18–22]. Importantly, AD neuropathology is known to develop decades prior to symptom onset, and factors like hyperglycemia and insulin resistance, which are cornerstones of diabetes pathophysiology, and associated with dementia and AD [23,24], may begin to confer risk in early life among individuals with youth-onset diabetes.

Advances in the aging and AD fields provide new opportunities to study the early life impact of diabetes on the risk of cognitive impairment and its underlying neuropathology. Specifically, measuring biomarkers of neurodegeneration, including glial fibrillary acidic protein (GFAP) and neurofilament light chain (NfL) protein, and of AD neuropathology, including phosphorylated Tau-181 (pTau-181), and amyloid beta (Aβ40 and Aβ42) in plasma is an efficient and non-invasive method to study the early pathologic risk of cognitive impairment and AD. GFAP, NfL, pTau-181, Aβ40, and Aβ42 are shown to be significantly associated with AD brain and cognitive alterations, as well as having good predictive value for identifying people at risk of cognitive impairment and AD prior to clinical symptoms [25–29]. Thus, measuring these biomarkers in youth-onset diabetes in conjunction with cognitive testing, both cross-sectionally and longitudinally, could provide much needed information about the potential for an increased and accelerated risk of cognitive impairment and AD in this highly vulnerable group.

The current study sought to address the significant gaps in our knowledge of the possible link between youth-onset diabetes and neurodegeneration and AD neuropathology by exploring the following: (1) differences in plasma-based biomarkers of neurodegeneration and AD neuropathology between adolescents and young adults with youth-onset diabetes and age-similar adolescent and young adult controls without diabetes; (2) changes across time in plasma-based biomarkers among adolescents and young adults with youth-onset diabetes; (3) the relationship between change over time in plasma-based biomarkers and cognitive function in young adulthood among people with youth-onset diabetes; and finally, (4) differences in gold-standard, clinical molecular imaging biomarkers of AD neuropathology between young adults with youth-onset diabetes and young adult controls without diabetes.

## 2. Materials and Methods

### 2.1. Participants with Youth-Onset Diabetes (Y-DM)

Participants with Y-DM (age at onset <20 years-old), T1D or T2D, were included from the SEARCH for Diabetes in Youth Study, a multicenter population-based registry and cohort. The design and study population of the SEARCH for Diabetes in Youth study have been extensively described elsewhere [30–32]. Briefly, incident cases of youth-onset T1D and T2D from the SEARCH Registry identified in 2002–2006, 2008, and 2012 were invited to participate in the SEARCH longitudinal cohort study that included a baseline research visit and several in-person follow-up visits conducted over 4 phases of data collection, SEARCH-1 (within 1 year of diabetes diagnosis in childhood and adolescence) through SEARCH-4 (follow-up in young adulthood). Each phase included the collection of health history, demographics, health-care related variables, clinical information, diabetes risk factors, and early diabetes-related complications and comorbidities.

A randomly selected subset of 50 SEARCH participants (n = 25 T1D, n = 25 T2D) were identified to be included in the plasma biomarker analysis. Participants were eligible for inclusion if they had completed the baseline SEARCH visit and the SEARCH-4 follow-up visit at either the Colorado or South Carolina clinic sites with stored plasma available from each visit, had etiologic defined T1D or T2D, were >25 years-old at SEARCH-4, had an average HbA1c > 75 mmol/mol (9%), had a confirmed presence of retinopathy and/or microalbuminuria at the SEARCH-4 follow-up visit, and had completed the cognitive testing battery at the SEARCH-4 follow-up visit. Of note, participants were selected for retinopathy and microalbuminuria given the evidence that microvascular complications may increase the risk of cognitive dysfunction in youth and adults with diabetes [19,33–36]. Participants were excluded if consent was not given for the use of stored samples (n = 3 T2D), leaving a total of 47 participants with Y-DM for plasma biomarker analyses.

Among the SEARCH participants eligible for the plasma biomarker analyses, we recruited and enrolled a subset from the Colorado SEARCH clinic site to complete positron emission tomography (PET) imaging to measure the accumulation of amyloid and tau density in AD-sensitive brain regions. Participants were excluded from imaging if they reported a major psychiatric disorder (e.g., schizophrenia, bipolar disorder, or major depression); a neurological condition affecting cognition (e.g., epilepsy), head trauma with loss of consciousness greater than 30 min, or stroke. Females were excluded if they were or planned to become pregnant. Additionally, participants with liver or kidney dysfunction were excluded from PET imaging procedures (estimated glomerular filtration rate [eGFR] < 45 mL/min per 1.73 m^2^ and/or serum albumin < 3.5 g/dL). All study procedures were approved by the Colorado Multiple Institution Review Board (COMIRB) and all participants provided written informed consent.

### 2.2. Control Participants

For the comparison of plasma-based biomarkers of neurodegeneration and AD, we identified age-similar controls without diabetes from two cohorts with stored plasma samples to include adolescent controls from the Exploring Perinatal Outcomes in Children (EPOCH) study (n = 25) and young adult controls from the Control of Renal Oxygen Consumption, Mitochondrial Dysfunction, and Insulin Resistance (CROCODILE) study (n = 21).

For the comparison of molecular imaging biomarkers of AD, we recruited and enrolled a group of young adult controls from the University of Colorado Anschutz Medical Campus to complete the PET imaging for amyloid and tau density in AD-sensitive brain regions. Participants were excluded similarly to the SEARCH subset for the reasons listed above. All study procedures were approved by COMIRB, and all participants provided written informed consent.

### 2.3. National Institutes of Health Toolbox Cognition Battery (NIHT-CB)

As described in detail previously [36,37], the NIHTB-CB was used to assess cognitive function at the SEARCH-4 follow-up visit in young adulthood. The NIHTB-CB assesses individual fluid and crystallized cognitive subdomains and derives composite scores for overall fluid and crystallized cognition that represent performance across all subdomain tests [38]. Subdomains of fluid cognition included cognitive flexibility (Dimensional Card Sorting Test), working (List Sorting Working Memory) and episodic (Picture Sequence Memory) memory, processing speed (Pattern Comparison Speed Test), and attention/inhibitory control (Flanker Inhibitory Control and Attention Test). In the present study, our primary cognition measure was composite fluid cognition in young adulthood. Age-corrected standard-scores based upon the normative population were used for the fluid composite score (mean = 100, SD = 15).

### 2.4. Plasma-Based Biomarkers of Neurodegeneration and AD Neuropathology

Plasma samples for each of the groups analyzed were collected as follows: SEARCH baseline samples (Y-DM adolescent) were collected in 2003–2010, with a mean [SD] storage duration of 17 years [3]; SEARCH-4 samples (Y-DM young adults) were collected in 2016–2019, with a mean [SD] storage duration of 4 years [1]; EPOCH samples (control adolescent) were collected in 2006–2015, with a mean [SD] storage duration of 9 years [3]; and CROCODILE samples (control young adults) were collected in 2020–2022, with a mean [SD] storage duration of 1 year [0.6].

Stored (−80 °C; polypropylene tubes) fasting plasma samples were assessed for biomarkers of AD via the Quanterix Simoa platform [39], including the Neurology 4-Plex A Advantage Kit measuring Aβ40, Aβ42, GFAP, and NfL, and the pTau181 Advantage V2 Assay Kit for the additional measurement of pTau181. Samples were analyzed by the Quanterix Accelerator Laboratory and run in duplicate. All analyses were restricted to replicates with a coefficient of variation < 15%.

### 2.5. Positron Emission Tomography (PET) of Amyloid and Tau Density in the Brain

Young adult SEARCH and control participants completed an amyloid PET imaging visit and a tau PET imaging visit (NCT# 05350514; IND#161840), separated by a minimum of 6 months. All scanning procedures were completed on a General Electric Signa 3T PET-MR scanner at the University of Colorado Research Imaging Center in Aurora, CO. At the first PET imaging visit, 5 mCi of [18F]PI-2620 radiotracer was administered via single intravenous bolus, followed by an intravenous flush of 0.9% sodium chloride injection (total volume 10 mL). List mode emission PET data were collected over a 30 min period and reconstructed to 6 five-minute frames beginning 60 min after radiotracer administration. The MRI portion of the scan included a sagittal 3D accelerated MPRAGE/IRSPGR, sagittal 3D FLAIR, axial T2* EPI, and axial 3D pCASL. A total of 5 mCi of [18F]Flutemetamol (VizamylTM) was administered via a single intravenous bolus at the second PET imaging visit, with list mode emission PET data collected over a 20 min period and reconstructed to 4 five-minute frames beginning 60 min after radiotracer administration. The MRI portion of the scan at the second imaging visit included a sagittal 3D accelerated MPRAGE/IRSPGR only.

PET images were processed and analyzed via established pipelines [40,41] by the Radiology Informatics and Image Processing Laboratory at Wake Forest School of Medicine. Briefly, standard uptake value ratio (SUVR) images were generated based on mean uptake post injection and normalized by mean inferior cerebellar GM uptake [42,43]. PET images were automatically co-registered to the MR-acquired MPRAGE from the PET-MR scanner, and standard brain labels (AAL) were generated using an automated brain-mapping algorithm software (SPM, version 12 [www.fil.ion.ucl.ac.uk/spm/software/], accessed on 3 June 2023). Analyses of ROIs from the AAL atlas and the AD-meta ROI were performed on SUVR images in subject space before and after partial volume correction, using published methods to adjust for off-target signal in ROIs and for atrophy [42,43]. In the current analysis, the primary outcome was the SUVR value for the AD-meta-ROI, which included the bilateral entorhinal cortex, fusiform gyri, inferior temporal lobes, middle temporal lobes, hippocampi, and amygdala.

### 2.6. Statistical Analyses

Descriptive information is reported using means and standard deviations, medians and interquartile ranges, or frequency counts and proportions, as appropriate. Due to the non-normality of the distributions of the plasma-based biomarkers and molecular imaging biomarkers, we applied nonparametric tests to estimate differences in plasma levels between Y-DM and control groups, specifically the Mann–Whitney U test. Complete-case comparisons of plasma-based biomarkers between adolescence and young adulthood in participants with Y-DM were similarly analyzed using nonparametric tests, specifically the paired Wilcoxon signed-rank test. Change scores in levels of plasma-based biomarkers between adolescence and young adulthood among participants with Y-DM were derived by taking the difference between the young adulthood plasma value and the adolescence plasma value for each individual. We then estimated Spearman correlations between change in plasma levels and fluid composite scores from NIHTB-CB completed at the SEARCH-4 follow-up visit in young adulthood.

## 3. Results

General descriptions of participants included in the plasma biomarker and the molecular imaging biomarker analyses are summarized in Table 1. Among the 47 participants eligible for the plasma biomarker analysis, 42 had stored plasma available from the baseline adolescent visit and all (n = 47) had available stored plasma from the SEARCH-4 young adulthood visit. Plasma-based biomarkers were quantified in all 25 adolescent controls included from the EPOCH cohort and all 21 young adult controls from the CROCODILE cohort.

### 3.1. Plasma Levels of GFAP, NfL, pTau181, A_β_40, and A_β_42 among Adolescents and Young Adults with Y-DM Compared to Adolescents and Young Adults without Diabetes

**Figure 1.**
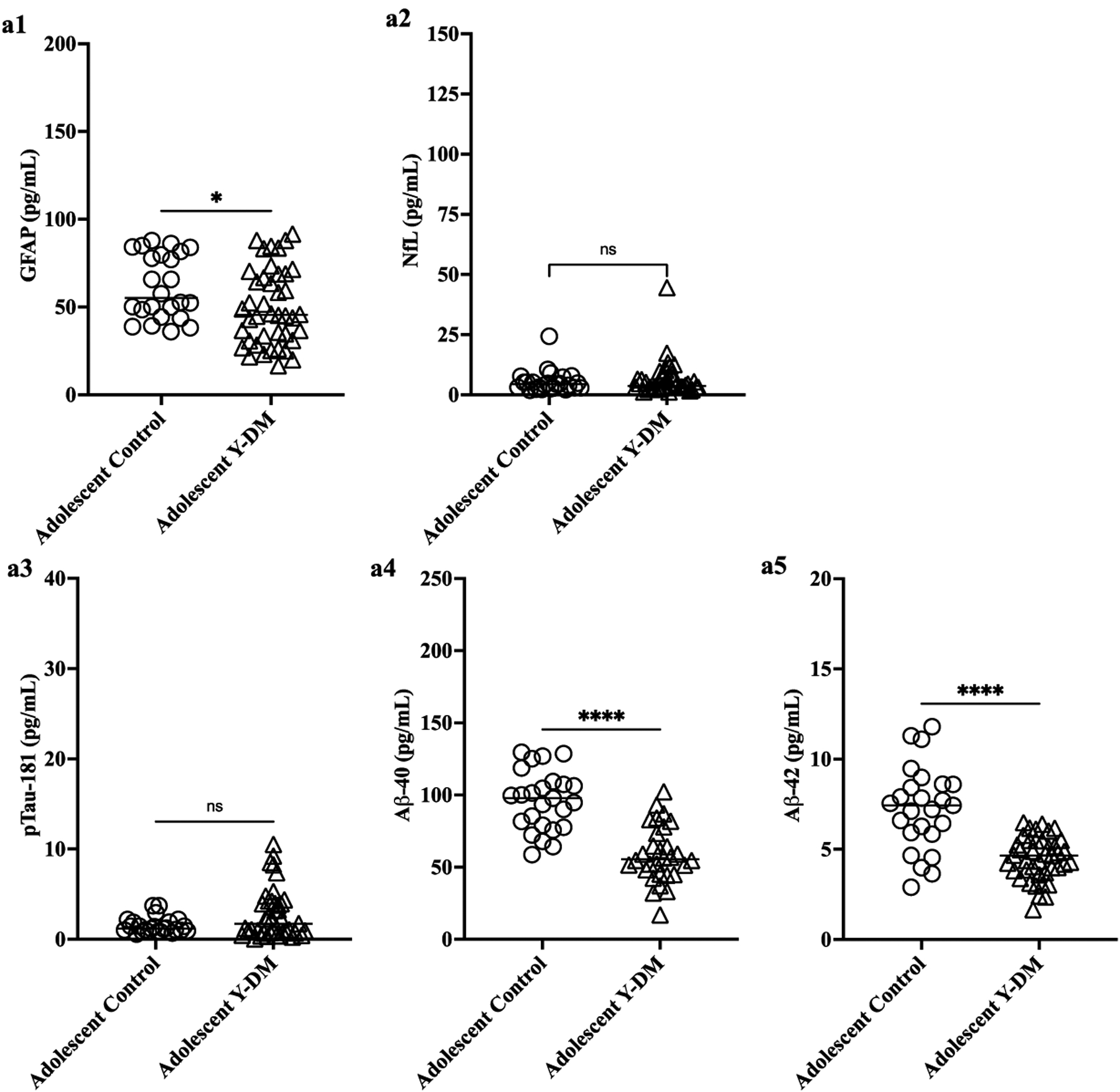

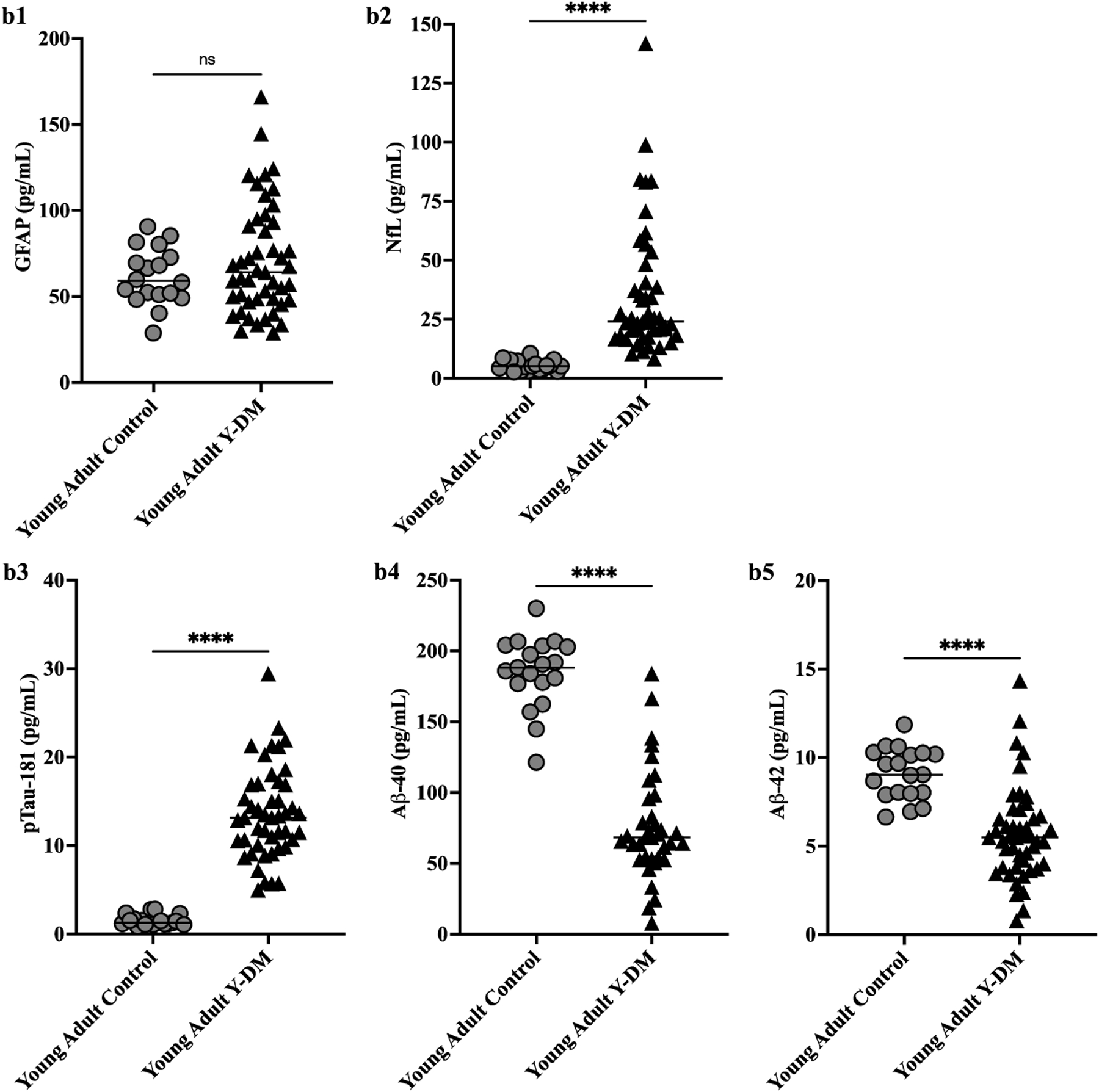
(**a**) Preclinical plasma-derived biomarkers of neurodegeneration (**a1**,**a2**) and AD neuropathology (**a3**–**a5**) in Y-DM (T1D and T2D) participants in adolescence versus age-similar adolescent controls. Biomarkers measured in stored plasma collected during adolescence and young adulthood. (**b**) Preclinical plasma-derived biomarkers of neurodegeneration (**b1**,**b2**) and AD neuropathology (**b3**–**b5**) in Y-DM (T1D and T2D) participants in young adulthood versus age-similar young adult controls. Biomarkers measured in stored plasma collected during adolescence and young adulthood. * *p* < 0.05; **** *p* < 0.001.

Figure 1a,b display plasma levels of each biomarker of neurodegeneration and neuroinflammation (GFAP, NfL) and AD neuropathology (pTau181, Aβ40, Aβ42) and their comparison between Y-DM and controls in adolescence and in young adulthood. Compared to age-similar controls, participants with Y-DM had significantly lower plasma Aβ40 (median [IQR]; 55.6 [45.2, 74.6] pg/mL vs. 97.8 [78.3, 108.3] pg/mL), Aβ42 (4.7 [3.8, 5.5] pg/mL vs. 7.4 [5.9, 8.6] pg/mL), and GFAP (45.5 [30.9, 68.8] pg/mL vs. 55.1 [45.4, 81.2] pg/mL), and higher pTau181 (1.7 [0.7, 4.1] pg/mL vs. 1.2 [0.9, 1.9] pg/mL) in adolescence (*p* < 0.05 for all). No differences were observed between groups in plasma levels of NfL (3.6 [3.1, 7.1] pg/mL vs. 4.4 [2.9, 6.3] pg/mL; *p* = 0.80).

Differences were similarly observed in young adulthood, where participants with Y-DM had significantly lower Aβ40 (68.4 [53.5, 92.6] pg/mL vs. 188.3 [177.0, 203.6] pg/mL) and Aβ42 (5.5 [3.8, 6.7] pg/mL vs. 9.0 [8.0, 10.3] pg/mL), and higher pTau181 (13.2 [10.1, 16.9] pg/mL vs. 1.3 [1.1, 1.7] pg/mL) and NfL (24.1 [18.2, 40.6] pg/mL vs. 5.2 [3.7, 7.3]), compared to age-similar controls (*p* < 0.001 for all). No differences were observed between these groups in plasma levels of GFAP (64.1 [48.2, 93.2] pg/mL vs. 59.1 [50.6, 74.7] pg/mL; *p* = 0.5). See Supplemental Figure S1 for biomarker levels in youth-onset T1D and T2D groups separated and compared individually to controls.

### 3.2. Correlations between Change in Plasma Levels of GFAP, NfL, pTau181, A_β_40, and A_β_42 from Adolescence to Young Adulthood and Cognitive Function in Participants with Y-DM

**Figure 2.**
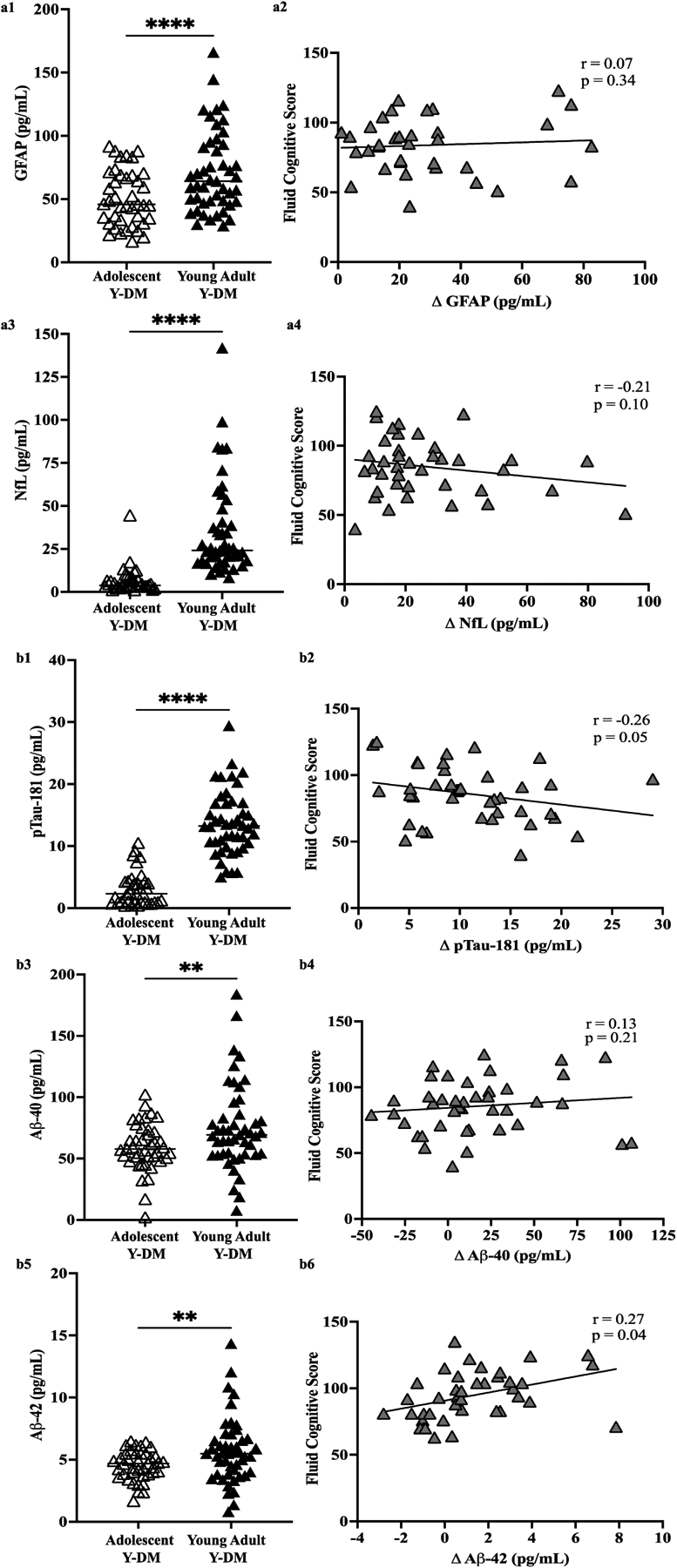
(**a**) Plasma levels of GFAP (**a1**,**a2**) and NfL (**a3**,**a4**) in adolescent Y-DM and their corresponding matched sample in young adulthood. (**b**) Plasma levels of pTau-181 (**b1**,**b2**), Aβ−40 (**b3**,**b4**), and Aβ−42 (**b5**,**b6**) in adolescent Y-DM and their corresponding matched sample in young adulthood. Mean values compared by paired *t*-test. Pearson correlations between change (Δ) in plasma values from adolescence to young adulthood in Y-DM (T1D and T2D) and overall fluid cognitive performance on the NIHTB-CB measured in young adulthood. ** *p* < 0.01; **** *p* < 0.001.

Figure 2a,b display plasma levels of each biomarker and their comparison between adolescence and young adulthood in Y-DM participants, and the correlation between change scores and fluid cognitive scores. Here, all plasma biomarkers increased significantly in participants with Y-DM from adolescence to young adulthood (*p* < 0.01 for all): GFAP (n = 41; median [IQR]; 45.8 [32.2, 68.9] pg/mL to 67.5 [47.5, 94.1] pg/mL), NfL (n = 38; 3.8 [3.2, 7.7] pg/mL to 24.8 [18.2, 42.6] pg/mL), pTau181 (n = 35; 2.3 [1.0, 4.3] pg/mL to 12.9 [9.7, 15.3] pg/mL), Aβ40 (n = 41; 57.8 [49.3, 72.7] pg/mL to 69.3 [53.5, 90.9] pg/mL), and Aβ42 (n = 40; 4.7 [3.8, 5.5] pg/mL to 5.5 [3.9, 6.6] pg/mL).

The change in plasma levels of Aβ42 across the approximately 12 years of follow-up was positively correlated with fluid cognitive scores (*p* = 0.04), that is, positive change (higher levels in young adulthood vs. adolescence) was related to higher fluid cognitive scores, whereas negative change (lower levels in young adulthood vs. adolescence) was related to lower fluid cognitive scores. The negative correlation between pTau181 change scores and fluid cognitive scores (r = −0.26) approached statistical significance (*p* = 0.05), with positive change related to lower fluid cognitive scores. No other correlations reached statistical significance.

### 3.3. PET Amyloid and Tau Accumulation in Young Adults with Youth-Onset Diabetes versus Young Adults without Diabetes

**Figure 3.**
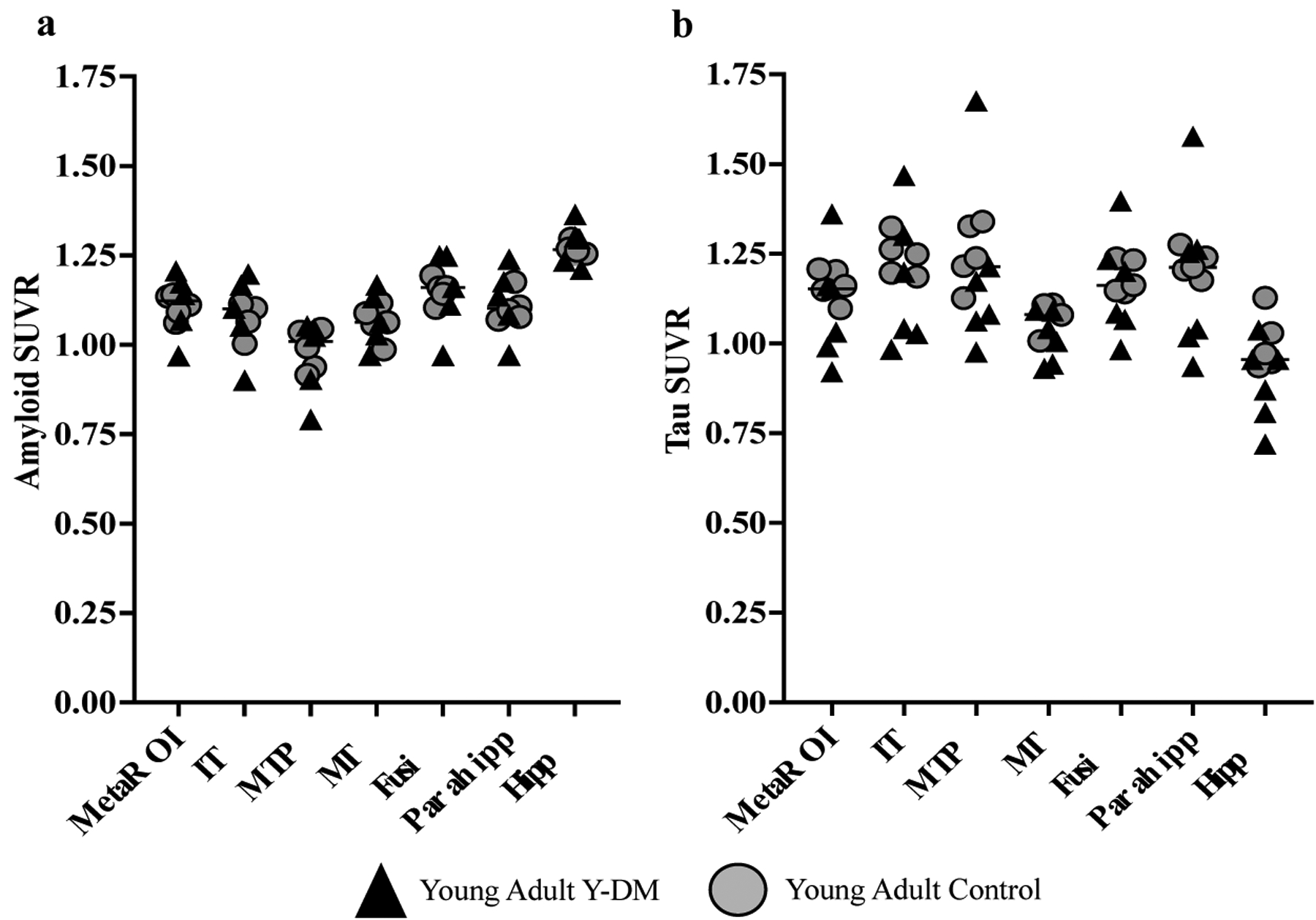
(**a**) PET amyloid (18F-Flutemetamol) and (**b**) tau (18F-PI2620) accumulation in AD meta-ROI and select AD-sensitive ROIs in young adults with Y-DM (T1D n = 5; T2D n = 1) and age-similar controls (n = 5). No statistically significant differences were found between groups. IT = inferior temporal; MTP = middle temporal pole; MT = middle temporal; Fusi = fusiform gyrus; Parahhip = parahippocampal gyrus; Hipp = hippocampal gyrus.

Figure 3 displays the amyloid and tau SUVRs for the meta-ROI and several example individual ROIs. On average, young adults with Y-DM had higher amyloid SUVR in the meta-ROI and the bilateral hippocampi, parahippocampi, and middle temporal poles, compared to age-similar controls. However, no differences reached statistical significance: meta-ROI (median [IQR]; 1.14 [1.07,1.17] vs. 1.11 [1.09, 1.13], *p* = 0.94), hippocampi (1.30 [1.23, 1.30] vs. 1.26 [1.25, 1.27], *p* = 0.62), parahippocampi (1.14 [1.08, 1.18] vs. 1.10 [1.08, 1.11], *p* = 0.76), fusiform gyri (1.16 [1.11, 1.25] vs. 1.16 [1.14, 1.16], *p* = 0.94), middle temporal gyri (1.06 [1.03, 1.13] vs. 1.06 [1.06, 1.09], *p* = 0.82), middle temporal poles (1.02 [0.90, 1.04] vs. 0.99 [0.94, 1.04], *p* = 0.71), and inferior temporal gyri (1.10 [1.05, 1.17] vs. 1.10 [1.06, 1.10], *p* = 0.90).

Tau SUVR was, on average, lower in young adults with Y-DM, compared to the age-similar controls in the meta-ROI and across all individual ROIs, although, again, no differences reached statistical significance: meta-ROI (median [IQR]; 1.15 [1.01,1.16] vs. 1.17 [1.14, 1.20], *p* = 0.41), hippocampi (0.95 [0.83, 0.96] vs. 0.99 [0.94, 1.05], *p* = 0.11), parahippocampi (1.21 [1.03, 1.26] vs. 1.22 [1.20, 1.25], *p* = 0.66), fusiform gyri (1.16 [1.07, 1.22] vs. 1.19 [1.15, 1.23], *p* = 0.63), middle temporal gyri (1.04 [0.97, 1.08] vs. 1.10 [1.07, 1.11], *p* = 0.18), middle temporal poles (1.17 [1.07, 1.28] vs. 1.23 [1.19, 1.26], *p* = 0.93), and inferior temporal gyri (1.20 [1.03, 1.25] vs. 1.25 [1.23, 1.28], *p* = 0.28).

## 4. Discussion

In this study. using plasma and molecular imaging biomarkers, we found evidence of potentially greater AD neuropathology in young adults with Y-DM, T1D and T2D, whereby plasma pTau181 was significantly higher and Aβ40 and Aβ42 were significantly lower, compared to controls, and over time from diabetes diagnosis in adolescence to young adulthood. Additionally, changes in key AD plasma biomarkers from the time of diabetes diagnosis to young adulthood were correlated with worse cognitive function in young adults with Y-DM. These preliminary data suggest the potential for an early-onset AD risk trajectory in people diagnosed with diabetes in childhood or adolescence.

While additional work is needed to replicate our findings, our results are comparable to the limited plasma biomarker studies in adults with diabetes. For example, in a matched case–control study in older adults with adult-onset T2D, Peters et al. (2017) observed lower plasma Aβ40 and Aβ42 in those with diabetes, compared to controls [44]. Similar to the increase over time in Aβ40 and Aβ42 observed in our Y-DM group, Hayden et al. (2024) also found an increase in these plasma biomarkers after 8 to 13 years of follow-up among middle age adults with T2D in the Look AHEAD trial [45]. However, despite using identical Quanterix platforms for plasma biomarker analysis, our Y-DM group had lower means of Aβ40 and Aβ42 (data not shown), compared to the means reported by Hayden et al. in adults with T2D [45]. Hayden et al. did not observe the nearly four-fold increase from baseline to follow-up in pTau181 among adults with T2D that we observed in the Y-DM group. Together, these data suggest that individuals with Y-DM are on a potentially accelerated AD risk trajectory, compared to people with adult-onset diabetes. However, comparisons to adult studies should be interpreted with caution, as the developmental significance of these biomarkers has not been extensively studied.

From our study, it is important to highlight that our participants with Y-DM had lower plasma concentrations of Aβ42 and Aβ40, compared to age-similar controls, during both adolescence and young adulthood, suggesting potentially early and sustained amyloid dysregulation in Y-DM. Lower levels of Aβ40, Aβ42, and their ratio, namely, in plasma and cerebrospinal fluid, correspond with monomer sequestration and the formation of amyloid plaques as measured by PET amyloid burden [46,47]. Overall, the lower plasma concentrations of Aβ42 and Aβ40 in our sample are suggestive of developing AD neuropathology but could also be indicative of disrupted neurodevelopment in people with Y-DM. Here, early Y-DM-related amyloid dysregulation may act as a “double hit” to diabetes-related cognitive dysfunction, thus, compounding the risk of early-onset cognitive impairment in people with Y-DM. Additional work is needed, however, to investigate the role of amyloid proteins in neurodevelopment in Y-DM.

Our study also found differences in plasma biomarkers of neurodegeneration, specifically GFAP, an indicator of neuroinflammation, and NfL, an indicator of neuron damage, between controls and Y-DM, and longitudinal changes across adolescence and young adulthood in Y-DM. In adolescence, Y-DM had lower average GFAP levels compared to adolescent controls, but in young adulthood, Y-DM had higher average GFAP as compared to young adult controls. While we would have expected GFAP to be higher in Y-DM adolescents, indicating some degree of diabetes-related neuroinflammation, the Y-DM group was newly diagnosed with youth-onset diabetes, where the average 1 year Y-DM duration prior to sample collection may not have been sufficient for measurable neuroinflammation to occur. Furthermore, the higher levels of GFAP in adolescent controls may not be indicative of neuroinflammation, and, in combination with the adolescent Y-DM group, may in fact be representative of the typical range of GFAP values in this age group.

On the other hand, the higher GFAP levels in the young adult Y-DM group, compared to the young adult controls, and the significant increase in GFAP levels in the Y-DM group between adolescence and young adulthood may represent diabetes-related neuroinflammation. This observation is consistent with other studies in adults with diabetes. In a recent study of older adults, Ayala-Guerrero et al. (2022) found significantly higher serum GFAP levels in people with T2D and cognitive impairment, compared to people without diabetes and who were cognitively unimpaired [48]. Interestingly, in this same study, serum GFAP levels followed a dose–response relationship where GFAP increased from cognitively unimpaired without T2D, to T2D only, to cognitively impaired only, and finally to T2D with cognitive impairment, suggesting that the presence of T2D confers added neuroimmune stress above and beyond that which underlies general cognitive impairment. Among our Y-DM group, the significant increase in plasma GFAP levels over the approximately 12-year follow-up period is also consistent with the Look AHEAD study, where they too observed significantly increased plasma GFAP levels in adults with T2D over an 8-to-13-year follow-up period [45].

Like GFAP, plasma NfL was higher on average in the young adult Y-DM group, compared to the young adult controls, but in diverging from the GFAP results, NfL was not different from controls during adolescence. NfL also significantly increased between adolescence and young adulthood in the Y-DM group. Again, these results are consistent with other, larger studies in adults with diabetes, where serum and plasma NfL are shown to be significantly higher, compared to nondiabetic reference groups, and increase with greater diabetes duration [45,49]. Despite this consistency, we exercise caution with the interpretation of our NfL results, given that NfL is a non-specific marker of neuron damage and could also indicate involvement from peripheral neuropathy in people with diabetes [50,51]. Overall, from the extant literature on GFAP and NfL in adult diabetes, we interpret our own results as evidence that some neuroinflammation and neuron damage is present and more pronounced in Y-DM and may be a direct result of diabetes. However, like our other findings, this too requires further investigation and replication in other cohorts.

In Y-DM, early and sustained cognitive dysfunction is well documented, especially among youth with T1D [52,53], and attributed, at least in part, to diabetes pathology including hyper- and hypoglycemia [54,55] and vascular complications [36,56]. Indeed, the Y-DM participants included in the current analysis were selected for inclusion based on their high HbA1c averages (hyperglycemia), and nearly all (94%) had mild retinopathy and approximately 50% had moderate albuminuria, a precursor to diabetes kidney disease. Remarkably, our results showed that a greater increase in plasma pTau181 and a greater decrease in plasma Aβ42 over an average of 12 years of follow-up was correlated with lower cognitive performance in young adults with Y-DM. Similar relationships have been observed with pTau181 in older adults, where higher pTau181 is associated with poorer baseline cognitive function and cognitive decline over time [57,58]. Increased pTau181 over time has also been shown to predict progression to AD dementia [59]. Decreasing plasma amyloid levels, particularly in the ratio of Aβ42-to-Aβ40, indicative of decreasing Aβ42 relative to Aβ40, are also associated with cognitive decline [60] and risk of AD dementia [60,61]. Thus, although primarily descriptive, our results are consistent with the extant literature in plasma biomarkers of AD and their relationship to cognitive function [62] and decline [63,64]. Interestingly, neither GFAP nor NfL change from adolescence to young adulthood was correlated with cognitive function. However, given the limitation of our single measure of cognitive function in young adulthood, further research is needed to examine how changes in biomarkers of neurodegeneration and AD relate to changes in cognition among youth and young adults with Y-DM.

Molecular imaging biomarkers of AD neuropathology are perhaps the least well established among people with diabetes, regardless of age. Contrary to the growing evidence of plasma-based AD neuropathology in adults with diabetes, the limited data on PET amyloid and tau are inconsistent. For example, in a recent study by Ennis et al. (2023), tau SUVR was not associated with diabetes status among a group of older adults from the Wisconsin Registry for Alzheimer’s Prevention [65]. However, in the Rotterdam cohort, van Arendonk et al. (2023) found that diabetes in older adults, which was confirmed at least 7 years prior to neuroimaging, was significantly associated with an increased risk of positive amyloid status via PET [66]. Still, others have found evidence to support more of a mixed neuropathology [67–70] underlying diabetes-related dementia. Our PET data neither support nor detract from the current body of evidence, given the (expected) absence of statistical differences in amyloid and tau SUVRs among young adults with Y-DM, compared to age-similar controls. One compelling interpretation of our plasma biomarker and cognitive results is that we have likely captured this group of individuals with Y-DM at early stages of neuropathological change and/or neurodegenerative damage, possibly via AD-related mechanisms. However, the intent of the PET study was hypothesis-generating, and future work in larger samples with longitudinal follow-up is needed to better understand brain amyloid and tau burden within the broader context of Y-DM and accelerated risk of dementia and AD.

### Strengths and Limitations

Although this study used a proof-of-concept design with a small sample size, our sample was representative of young people with Y-DM in the United States and included T1D, T2D, and age-similar controls, as well as validated AD plasma biomarkers and gold-standard AD molecular imaging biomarkers. Furthermore, within our group of young people with Y-DM, the longitudinal measurement of the plasma AD biomarkers ameliorated issues of reverse causality, such that the first measurement was taken from samples collected in adolescence, within 1 year of diabetes diagnosis, and the second in young adulthood after approximately 12 years of follow-up. Thus, we interpret these significant changes as the result of diabetes and related factors over the follow-up period. However, given that we selected age-similar controls from different cohorts (no longitudinal data), we do not know if similar changes would be seen in a repeated sampling of young people without diabetes. Importantly, the young age of our sample limits confounding by age, such that the changes in plasma biomarkers that we observed in Y-DM are more likely to be attributed to diabetes pathophysiology and not to typical aging-related processes.

While noting our study’s strengths, the results should be interpreted with caution given several limitations. First, we did not have APOE4 status among the people included in our study. Of note, however, a prior study showed that APOE4 status did not influence plasma levels of amyloid in young people without cognitive dysfunction or diabetes [71]. Second, the SEARCH study did not measure cognitive function at the baseline visit, and thus we were unable to investigate cognitive change over time relative to change in plasma biomarker levels. Third, we do not have corresponding biomarkers measured in CSF, which, in addition to the PET imaging of amyloid and tau, is considered a gold standard measure of AD neuropathology. Further, our sample of young adults with Y-DM who participated in the PET imaging study was too small to conduct a head-to-head comparison of plasma and molecular imaging biomarkers and estimate their concordance in Y-DM. Finally, unlike the longitudinal sampling of plasma available in the Y-DM group from the SEARCH cohort, our age-similar control groups were sampled from different cohorts. Thus, we cannot interpret the differences in biomarkers between the adolescent controls and the young adult controls as typical developmental change in these biomarkers. However, regardless of the control group, Y-DM youth and young adults differed from age-similar young people without diabetes.

It should also be noted that the storage duration of each groups’ plasma samples differed, imparting yet another limitation to our study, with Y-DM samples having longer storage duration on average, compared to control samples. Longer storage duration could impact the observed protein concentrations of the plasma biomarkers measured. Specifically, lower protein concentrations could reflect greater protein degradation because of longer storage duration, even at −80 °C. However, if protein levels were differentially impacted between groups given the variability in storage duration, we might expect to see lower concentrations in the Y-DM group compared to the control groups across all proteins measured, which was not the case in our study. Additionally, it has been shown that pre-analytic sample handling, specifically the material of the tube in which the plasma or CSF is stored, can affect protein levels of AD biomarkers, particularly Aβ42 and Aβ40 [72]. All samples used in the current study were stored in polypropylene tubes. Thus, if storage material impacted protein levels in our samples, the effect is likely to be consistent across all groups.

## 5. Conclusions

In conclusion, although AD is conceptualized as a late life disease, increasing evidence, including the results reported here, suggest that early life factors may impact risk trajectories. This work reinforces the necessity of applying life course models of risk of neurodegenerative diseases like AD [73]. Such life course models will help to facilitate a deeper understanding of how the AD biomarkers currently in use change during critical developmental periods across the life course, and how they may be used to predict the risk of early-onset neurodegenerative diseases and cognitive impairment in high-risk clinical populations like Y-DM.

## Supplementary Material

Figure S1

## Figures and Tables

**Table 1.** (**a**) General descriptors of participants included in the plasma-based biomarker adolescent and young adult analytic samples. (**b**) General descriptors of participants included in the molecular (PET) imaging biomarker analytic sample.

(a)				
	Adolescent Controls (n = 25)	Adolescent Y-DM (n = 42)	Young Adult Controls (n = 21)	Young Adult Y-DM (n = 47)
Age (years), mean (SD)	14.8 (2.7)	15.0 (2.6)	24.9 (2.8)	27.4 (2.2)
Sex (female), n (%)	15 (60)	24 (57)	10 (48)	28 (59)
Race and ethnicity, n (%)				
Hispanic	10 (40)	9 (21)	3 (14)	9 (19)
Non-Hispanic Black	4 (16)	20 (48)	1 (5)	22 (47)
Non-Hispanic White	10 (40)	12 (28)	16 (76)	15 (32)
Other or multiple race and ethnicity	1 (4)	1 (3)	1 (5)	1 (2)
Y-DM status, n (%)				
T1D	--	20 (48)	--	25 (53)
T2D	--	22 (52)	--	22 (47)
Diabetes duration (years), mean (SD)	--	1.0 (0.6)	--	13.6 (2.4)
Retinopathy (yes), n (%)	--	--	--	44 (94)
Microalbuminuria (yes), n (%)	--	--	--	23 (49)
(b)				
	Young Adult Controls (n = 6)		Young Adult Y-DM (n = 7)	
Age (years), mean (SD)	25.1 (4.5)		27.5 (5.7)	
Sex (female), n (%)	4 (67)		5 (71)	
Race and ethnicity, n (%)				
Hispanic	0		2 (28)	
Non-Hispanic Black	2 (33)		1 (14)	
Non-Hispanic White	2 (33)		2 (28)	
Other or multiple race and ethnicity	2 (33)		2 (28)	
Y-DM status, n (%)				
T1D	--		6 (86)	
T2D	--		1 (14)	

## Data Availability

Data are available on request from the authors and in accordance with consent given by participants and University of Colorado data sharing policies.
