# Supplementary figures and images for: Biomarkers of Neurodegeneration and Alzheimer’s Disease Neuropathology in Adolescents and Young Adults with Youth-Onset Type 1 or Type 2 Diabetes: A Proof-of-Concept Study
Source: Endocrines. Author manuscript; Available in PMC 2024 Jun 1. (PMC11101213; doi:10.3390/endocrines5020014)

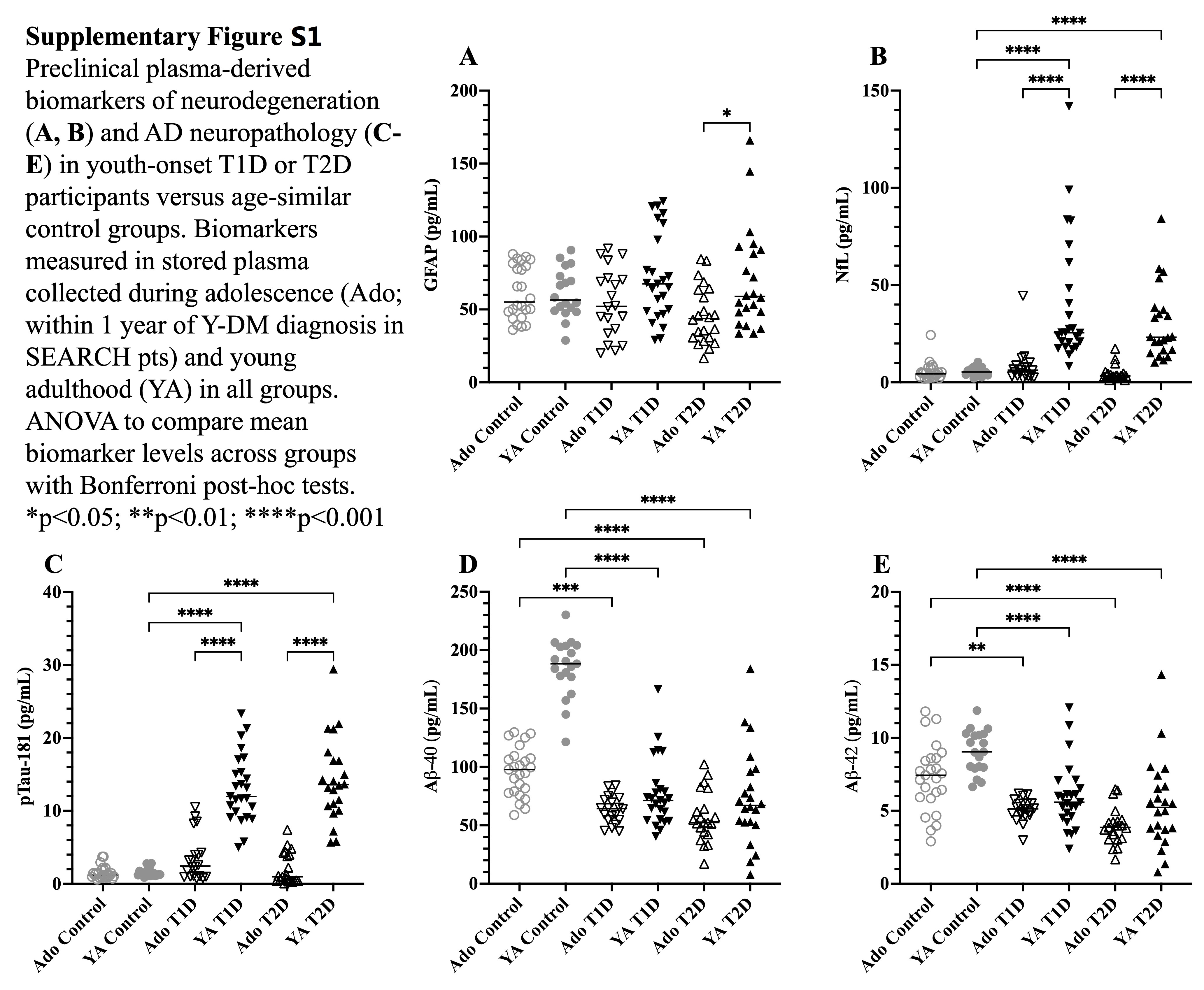

Supplement: Figure S1 [file NIHMS1991998-supplement-Figure_S1.jpg]
